# Dataset size considerations for robust acoustic and phonetic speech encoding models in EEG

**DOI:** 10.3389/fnhum.2022.1001171

**Published:** 2023-01-20

**Authors:** Maansi Desai, Alyssa M. Field, Liberty S. Hamilton

**Affiliations:** ^1^Department of Speech, Language, and Hearing Sciences, Moody College of Communication, The University of Texas at Austin, Austin, TX, United States; ^2^Department of Neurology, Dell Medical School, The University of Texas at Austin, Austin, TX, United States

**Keywords:** electroencephalography, linear model, natural stimuli, spectrotemporal receptive field, experimental design

## Abstract

In many experiments that investigate auditory and speech processing in the brain using electroencephalography (EEG), the experimental paradigm is often lengthy and tedious. Typically, the experimenter errs on the side of including more data, more trials, and therefore conducting a longer task to ensure that the data are robust and effects are measurable. Recent studies used naturalistic stimuli to investigate the brain's response to individual or a combination of multiple speech features using system identification techniques, such as multivariate temporal receptive field (mTRF) analyses. The neural data collected from such experiments must be divided into a training set and a test set to fit and validate the mTRF weights. While a good strategy is clearly to collect as much data as is feasible, it is unclear how much data are needed to achieve stable results. Furthermore, it is unclear whether the specific stimulus used for mTRF fitting and the choice of feature representation affects how much data would be required for robust and generalizable results. Here, we used previously collected EEG data from our lab using sentence stimuli and movie stimuli as well as EEG data from an open-source dataset using audiobook stimuli to better understand how much data needs to be collected for naturalistic speech experiments measuring acoustic and phonetic tuning. We found that the EEG receptive field structure tested here stabilizes after collecting a training dataset of approximately 200 s of TIMIT sentences, around 600 s of movie trailers training set data, and approximately 460 s of audiobook training set data. Thus, we provide suggestions on the minimum amount of data that would be necessary for fitting mTRFs from naturalistic listening data. Our findings are motivated by highly practical concerns when working with children, patient populations, or others who may not tolerate long study sessions. These findings will aid future researchers who wish to study naturalistic speech processing in healthy and clinical populations while minimizing participant fatigue and retaining signal quality.

## 1. Introduction

The use of naturalistic stimuli has become increasingly popular when investigating speech tracking in the brain using EEG. A benefit of using naturalistic stimuli is that it is possible to fit linear regression models to map the relationship between specific speech features and the brain data. These models are commonly known as an “encoding model” or “forward model” (Aertsen and Johannesma, 1981; Wu et al., 2006; Crosse et al., 2016; Holdgraf et al., 2017). Such models allow researchers to estimate a multivariate temporal receptive field [mTRF, also called multivariate temporal response function (Crosse et al., 2016)], which describes the brain's time varying response to combinations of acoustic or linguistic features of interest. To fit these models, researchers have used audiobooks, speech corpora such as the Texas Instruments Massachusetts Institute of Technology (TIMIT) (Garofolo et al., 1993), and children's movie trailers (Desai et al., 2021) to predict neural responses to the acoustic envelope, pitch, spectrogram, phonological features, and semantic features (Mesgarani et al., 2014; Di Liberto et al., 2015; Khalighinejad et al., 2017; Tang et al., 2017; Brodbeck et al., 2018; Hamilton et al., 2018; Teoh et al., 2019; Desai et al., 2021).

A major gap in the current literature is that the amount of training and testing data needed for building robust encoding models is unknown. Especially in cases where data collection time may be limited or cut short, experimenters should understand whether and how results will differ based on the amount of available data. On the one hand, natural stimulus paradigms differ from traditional event related potential (ERP) analyses in that they do not require presentation of the same stimulus many hundreds of times, but rather, rely on repetition of acoustic or linguistic elements within the natural stimulus itself (Luck, 2014; Crosse et al., 2016). Because of this fundamental difference in the overall design of such experiments, it can be unintuitive to understand the relative data requirements for a study using forward modeling as compared to an ERP study. Recent papers discuss the benefits and methodology of using linear regression modeling to build temporal receptive fields (TRFs) models and linear decoders, in which a common suggestion is to use as much data as possible (Crosse et al., 2016; Holdgraf et al., 2017). While this may be true to a certain extent, the authors point out that collecting human brain data is time restrictive. Conducting pilot studies can present experimenters with a ballpark idea to assess the optimal number of trials needed. From this, one can calculate a learning curve to identify when adding more training data no longer improves model performance (Holdgraf et al., 2017). Ultimately, one could stop collecting training data once the model is “stable” (Willmore and Smyth, 2003).

Crosse et al. (2016) briefly describes collecting a minimum of 10–20 min of data for each condition, reiterating that collecting as much data as possible is ideal. However, a caveat with human data collection is participant fatigue, particularly when working with clinical populations or children. Although not a major focus here, working with intracranial EEG in patients with epilepsy can be extremely time-limited, so planning of tasks and understanding how much data is required for each task is paramount (Miller et al., 2021). If, for example, a researcher can acquire 10 min of data (but no more) for a given task, are the data usable? Additionally, the amount of data required may depend on the feature representation used in the regression model–how many features, how many samples of each feature are available, covariance/autocorrelation within the features, and more. Fitting a model using multivariate regression models may require more data for features that are sparse (Crosse et al., 2016; Mesik and Wojtczak, 2022). While several suggestions exist, such as splitting up the trials into multiple recording sessions, this still does not address the question of the minimal amount of training data required when building an experiment for pediatric or clinical populations. Collecting as much data as when possible is the most ideal and preferable. However, there may be situations when researchers are only able to collect a minimal amount of data. Here, we provide methods to assess whether such data may be usable and whether receptive field results are stable.

In this paper, we use data from two previously reported EEG experiments (Broderick et al., 2018; Desai et al., 2021) and provide some suggestions on approximately how much data are needed to fit robust receptive field models for different stimuli and different feature sets. In our prior work (Desai et al., 2021), we fit encoding models on individual auditory features (e.g., phonological features, the acoustic envelope, and pitch) and a combination of these features using two contrasting datasets: TIMIT and movie trailers. While TIMIT and movie trailers are not widely used stimuli for typical natural speech experience for EEG, audiobooks have shown to generate robust encoding model performance. Thus, in addition to previously collected natural speech stimuli from our laboratory, we also incorporated an open EEG dataset from a different study to provide more insight about generalizability to other datasets (Broderick et al., 2018). Here, we provide suggestions in assessing the amount of training data needed if one were to use a single feature representation (such as phonological features or the acoustic envelope or pitch) for specific stimuli vs. using a combination of all features (phonological features plus the acoustic envelope plus pitch). As a part of our broader goal, identifying the amount of training data for natural speech experiments would be immensely beneficial in minimizing participant fatigue for both healthy participants (those without hearing impairments or neurological issues), clinical populations, and children.

## 2. Materials and methods

We incorporated EEG data from our own previous work (Desai et al., 2021) as well as the work of Broderick et al. (2018). For the first dataset, the data, experimental paradigm, and participants are the same as the methods described in Desai et al. (2021). Data collection and preprocessing are described in detail in the aforementioned paper. All procedures were approved by The University of Texas at Austin Institutional Review Board. All participants provided their written informed consent to participate in this study.

In brief, 16 participants (8M, age 20–35) listened to two sets of stimuli while scalp EEG was acquired. These stimuli included sentences without noise, taken from the Texas Instruments Massachusetts Institute of Technology (TIMIT) corpus (Garofolo et al., 1993) and children's movie trailers, which were hand transcribed for word and phoneme level boundaries by undergraduate research assistants in the laboratory (Desai et al., 2021). We collected 64-channel high density scalp EEG data (Brainvision actiChamp System) at a sampling rate of 25 kHz. The stimuli were delivered through insert ear buds **(**3M, E-A-Rtone Gold 10Ω, Minnesota, USA**)** and were recorded through a StimTrak system (BrainProducts), which directly synchronizes with the recorded EEG signal in a separate audio channel. Vertical and horizontal electrooculography electrodes were placed to capture any ocular artifact from blinks and saccades, respectively.

For the second dataset, we included freely available natural speech listening EEG data from Broderick et al. (2018) (available at https://datadryad.org/stash/dataset/10.5061/dryad.070jc, “Natural Speech” dataset). In that study, 19 native English-speaking participants (13 M, age 19–38 years) listened to ~60 min of the audiobook “*The Old Man and the Sea”* while 128-channel scalp EEG was recorded. For all 19 subjects, preprocessing was similar to the steps conducted from Desai et al. (2021). Neural data were notch filtered at 50 Hz and then filtered between 1 and 15 Hz. Data segments were manually inspected for motion artifact and the respective time segments were subsequently rejection. ICA was finally conducted to identify and remove ocular artifacts.

### 2.1. Stimuli

A total of 380 TIMIT sentences were presented to all of our EEG participants. Out of the 380 sentences, 10 of the sentences were repeated 10 times and the average neural response of these 10 sentences was used as the test set. The remaining 370 TIMIT sentences were used in the training set. A total of 23 movie trailers (Supplementary Figure 1) were presented. In all except three subjects, two of the trailers that were heard twice (Inside Out and Paddington 2) were used as the test set. In the case where one of the test set stimuli was not heard for three of the subjects, the test set consisted of just one of the trailers heard (either Inside Out or Paddington 2).

In addition to the TIMIT and movie trailer EEG data we collected in our laboratory, we included an analysis of a separate 128-channel EEG dataset from Broderick et al. (2018). In this study, the authors presented audiobooks to EEG subjects and fit linear encoding models to understand the neural tracking of semantic information and how such higher-order information relates to lower-level feature representations.

### 2.2. EEG preprocessing

For the TIMIT and movie trailer data, EEG data were downsampled to 128 Hz and bandpass filtered between 1 and 15 Hz using a zero-phase, non-causal bandpass finite impulse response filter (Hamming window, 0.0194; passband ripple with 53 dB stopband attenuation, −6 dB falloff). Data were notch filtered at 60 Hz and manual artifact rejection was performed to remove any non-biological artifacts which may have occurred during recording (e.g., movement, electromyography). Ocular artifacts were removed using independent component analysis (ICA) and correcting for components with clear blink or saccade-like topography. Audio stimuli were synchronized with the EEG data using a customized match filter script (Turin, 1960) in which the audio signal was convolved with the stimuli presented to detect the onset and offset of each stimulus. Aligned neural responses for each of the 64 scalp electrodes and stimulus matrices with acoustic envelope, pitch, and phonological features were used as inputs to the model. We included 14 phonological features that encompassed both place and manner of articulation features (sonorant, obstruent, voiced, back, front, low, high, dorsal, coronal, labial, syllabic, plosive, fricative, nasal) and have been used in prior work (Mesgarani et al., 2014; Hamilton et al., 2018; Desai et al., 2021). The onset of each of these features was coded as a binary stimulus matrix. The acoustic envelope of each speech stimulus was extracted using the Hilbert transform followed by a lowpass filter (3rd order Butterworth filter, cut off frequency 25 Hz). The pitch was calculated as the fundamental frequency (f0) of each stimulus using the PraatIO package in python (Jadoul et al., 2018).

For the audiobook data, preprocessing was the same as the methods described above for EEG data collected in our laboratory. To complement the lower-level acoustic feature encoding described in the analysis section of this manuscript, we used the acoustic envelope information stored in the dataset alongside the raw EEG data.

### 2.3. Analysis

As in prior work, we fit encoding models using a linear regression approach, which we refer to as a multivariate temporal receptive function (mTRF) (Theunissen et al., 2001; Mesgarani et al., 2014; Hamilton et al., 2018; Desai et al., 2021). The fundamental goal is to quantify the relationship between the auditory feature (input) and the predicted EEG response at a specific time for a given feature (output). We fit separate models for all 64 EEG channels. This allowed us to evaluate how the brain tracks specific speech features while investigating how much training data was needed to achieve maximal prediction performance from the linear model. The equation for our encoding model is as follows:


$$\begin{array}{l} EEG\left(t,n\right)=\;\underset{f}{\sum }\underset{\tau }{\sum }w\left(f,\tau ,n\right)s\left(f,t-\tau \right)+\;\varepsilon \left(t,n\right) \end{array}$$

EEG (*t,n*) represents the measured bandpass filtered EEG at time t for electrode n, *w*(*f*, τ, *n*) is a matrix of fitted weights for each feature *f* at time delay τ, and *s*(*f, t*−τ) represents the stimulus features at each time delay relative to the neural activity, and ε(*t, n*) is the residual error. For all models, we fit mTRFs using time delays from 0 to 600 ms, based on speech encoding models from previous work (Hamilton et al., 2018; Desai et al., 2021). We performed these analyses using customized scripts in Python that implemented cross-validated ridge regression. The weights (*w*) were calculated by first choosing a random subset of the training data, then fitting an encoding model on that subset of data. The size of the data subset started with 10 random sentences for TIMIT, or 10 2-s chunks of movie trailers or audiobook stimuli. This chunk size was chosen as it is similar to the average TIMIT sentence length. The test set for the movie trailers was the average neural response of two movie trailers each played twice. In contrast, for the audiobook data we used 80% of the data as the training set and 20% of the data as the test set. The performance of the model was tested using a held-out validation set, which was kept the same regardless of training set size. After fitting the models on a subset of data, the size of the training set was gradually increased (by 1 random TIMIT sentence at a time or a random 2-s chunk of movie trailers or a random 2-s chunk of audiobooks). For each training set size, we used a 10-fold bootstrapping procedure so that the particular 2-s chunks included in the training set were sampled randomly from the original training dataset. For all regression analyses, the ridge regularization parameters (alphas) on models previously fit on all data for the stimulus type (TIMIT or movie trailers) and the specific feature representations on a subject-by-subject basis. Since no previous models were fit in our laboratory using the audiobooks, we tested 15 alpha values between 10^2^ and 10^8^ based off the regularization parameterization from Desai et al. (2021).

We then assessed the model performance (correlation between predicted and actual held out data) as a function of amount of training data. The correlation value was averaged after calculating the correlation of each EEG electrode separately. Results were then plotted on a per-subject basis to demonstrate how the average correlation value changes as a result of adding increasing amounts of training set data. To determine the amount of data at which this performance starts to plateau, we calculated a knee point (Satopää et al., 2011) for each individual auditory feature or combination of features. This was performed separately for models trained on TIMIT, movie trailer, or audiobook data. The knee point was calculated for each subject and averaged together to represent a grand average knee point. A knee point calculation was found for all subjects except for one (MT0001) for the movie trailer, three subjects (MT0013, MT0014, and MT0017) for the TIMIT stimuli, and one subject (Subject11) for the audiobooks. We used the following equation to define the curvature of the correlation *f* (*x*) vs. the number of training samples (*x*) (Satopää et al., 2011).


$$K_{f}(x)=\frac{f^{\prime \prime }(x)}{{\left(1+f^{\text{'}}{\left(x\right)}^{2}\right)}^{1.5}}$$

From this equation, we took the value of *x* for which the curvature *K* is maximized as the “knee point.”

To compare whether the knee point of this correlation curve was significantly influenced by the model type, we used linear mixed effects models (LME) implemented in R using the library lmerTest (Kuznetsova et al., 2017) and emMeans (Searle et al., 1980) to calculate the estimated marginal means. We used the knee point calculation for each model type (phonological features only, envelope only, pitch only, or full model with all features) as a fixed effect, and participant intercept as a random effect. This took the form of the following LME equation (Yu et al., 2021): knee point ~ model + (1|subject). *P*-values and degrees of freedom (*df*) were estimated using Satterthwaite's method.

## 3. Results

### 3.1. How much training data are needed for a combination of auditory features for both TIMIT and movie trailers?

**Figure 1:**
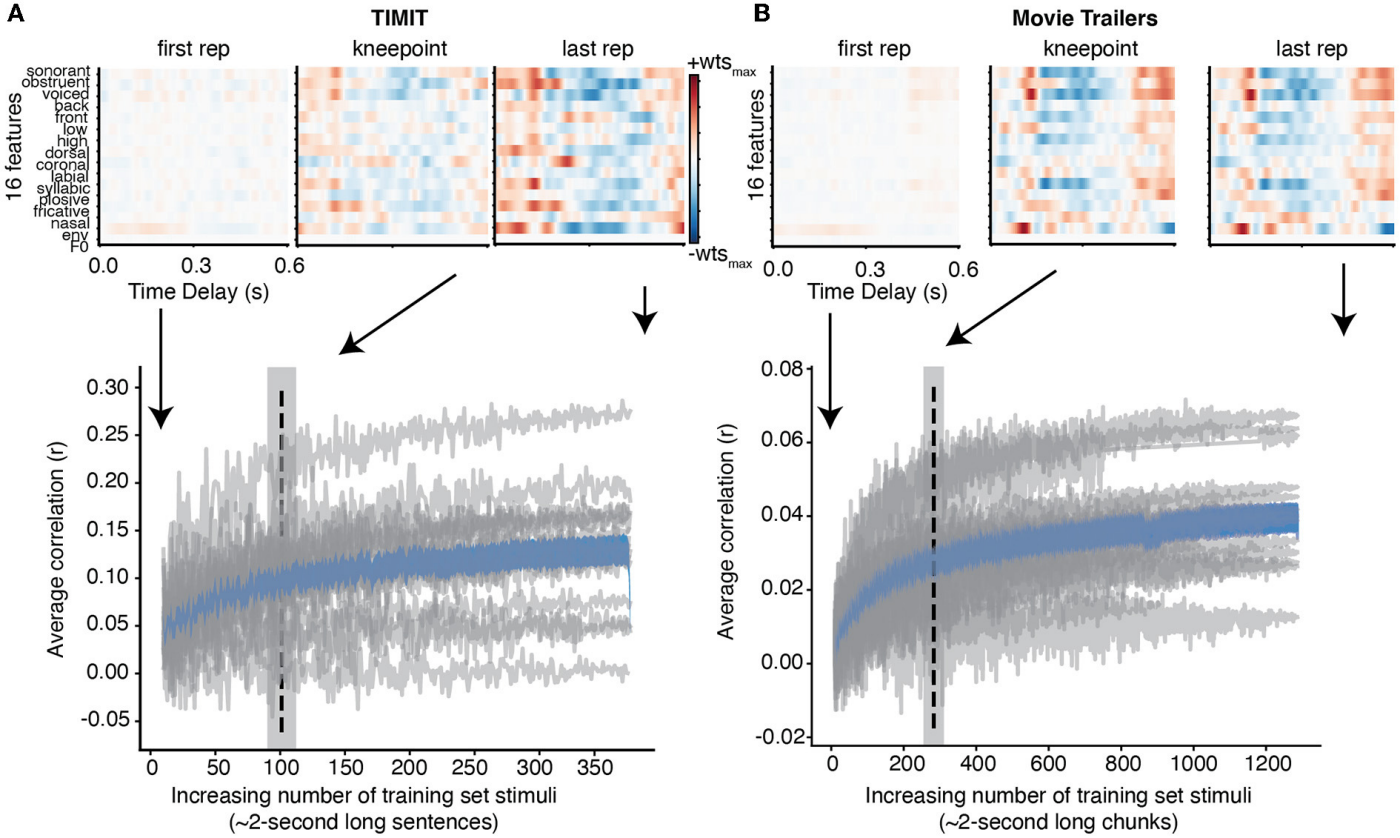
Individual and average correlation values with increasing training data. **(A)** For TIMIT, each gray line shows how the correlation values change for an individual subject when adding each additional TIMIT sentence to the training data. The average increasing correlation value of all subjects is shown in blue with standard error. The dashed vertical line at 96 sentences indicates the average knee point across all subjects (gray shading shows standard error of the mean). Finally, the weights for the full model for a single subject with the highest correlation values (MT0008) is shown at the first repetition (e.g., when there is only one TIMIT sentence to train on), the knee point, and the final repetition (e.g., after adding all TIMIT sentences specified in the training set). The structure of the receptive field at the knee point and at the last repetition is qualitatively similar. **(B)** Same as TIMIT but for movie trailers. Again, the receptive field at the knee point (with only 565.75 s of training data) is qualitatively similar to the receptive field at the last repetition (2,554 s).

We first assessed how much training data were needed for encoding models across all subjects for both the TIMIT and movie trailer stimuli. We fit an encoding model using a combination of all three auditory features (phonological features, acoustic envelope, and pitch; henceforth called the “full model”) to predict the neural activity at each iteration of adding training data. In addition, we fit an encoding model using just the acoustic envelope for the audiobook data as this was the only low-level feature representation included with the dataset. This analysis was performed for each individual subject, and a correlation (*r*-value) was computed for each iteration based on the predicted versus actual brain response from the encoding model with a subset of the training data for TIMIT movie trailers, and audiobooks (Figure 1). Individual participants are shown in gray, with the average correlation vs. the data size curve shown in blue. While the amount of training data needed in the model plateaus as evidenced by this average, some individual participants show a clear increase in performance as data are added, while others do not improve with added data.

The knee point was calculated for each individual subject and then averaged together (dashed line). This knee point shows the time at which performance begins to plateau; however, it does not show whether the receptive field structure itself changes substantially as data are added. To visualize this process, we plotted the weights for the subject with the best model performance (MT0008) for both TIMIT and movie trailers at the first repetition, the knee point, and at the last repetition (linear regression correlation values (See Supplementary Video 1). In the case of both TIMIT and movie trailers, the receptive field weights at the first repetition are relatively weak and lack structure, suggesting that additional training data are needed to achieve robust model performance. For TIMIT, the weight magnitude at the knee point was slightly lower than at the last repetition, but the structure of the weights themselves were largely similar. In contrast, the knee point and last repetition for the movie trailers were visually similar, suggesting that the weights for MT0008 are relatively stable even with increasing amounts of training data up until the final repetition. An average of 96 sentences of TIMIT data (192 s) were needed to reach stable performance when using a combination of all phonological features, envelope, and pitch (Figure 1A).

For the movie trailers, we found that an average of 565.75 s (or 9.43 min) were needed for to reach stable performance (Figure 1B). Of note, the overall correlation values for movie trailers are much smaller compared to TIMIT, which is consistent with results from our previous work (Desai et al., 2021). As we have previously described, models using the movie trailers as the stimuli can still provide generalizable receptive field results (Desai et al., 2021). However, a reason for the lower correlation values between the actual and predicted EEG response is likely attributed to the overlapping sound sources of multiple talkers, background noise, and music compared to the sentences present in isolation during the TIMIT condition.

For the audiobooks, we found that an average of 495.47 s or (8.23 min) were needed to reach stable performance across the 19 EEG subjects (Supplementary Figure 2). The correlation values were much smaller compared to TIMIT and movie trailers. A possibility for these lower correlation values may be that single trials were used rather than trial averages in the test set. Nonetheless, the knee point performance and correlation values demonstrate a similar time course to our own EEG data.

Finally, to investigate the effect of rank based on the EEG channels for all stimuli, we calculated the rank using a method which takes into account biological systems because recording human data from sensors has variability in the signal-to-noise ratio (Litwin-Kumar et al., 2017). Traditional rank calculation methods do not account for varying underlying noise and may overestimate data rank. We found that the relationship between rank and knee point was not correlated for either the movie trailer or TIMIT stimuli. For 3 subjects who listened to the TIMIT sentences and for 1 subject who listened to movie trailers, we were unable to calculate a knee point.

### 3.2. How much training data are needed based on stimulus type and feature representation?

**Figure 2:**
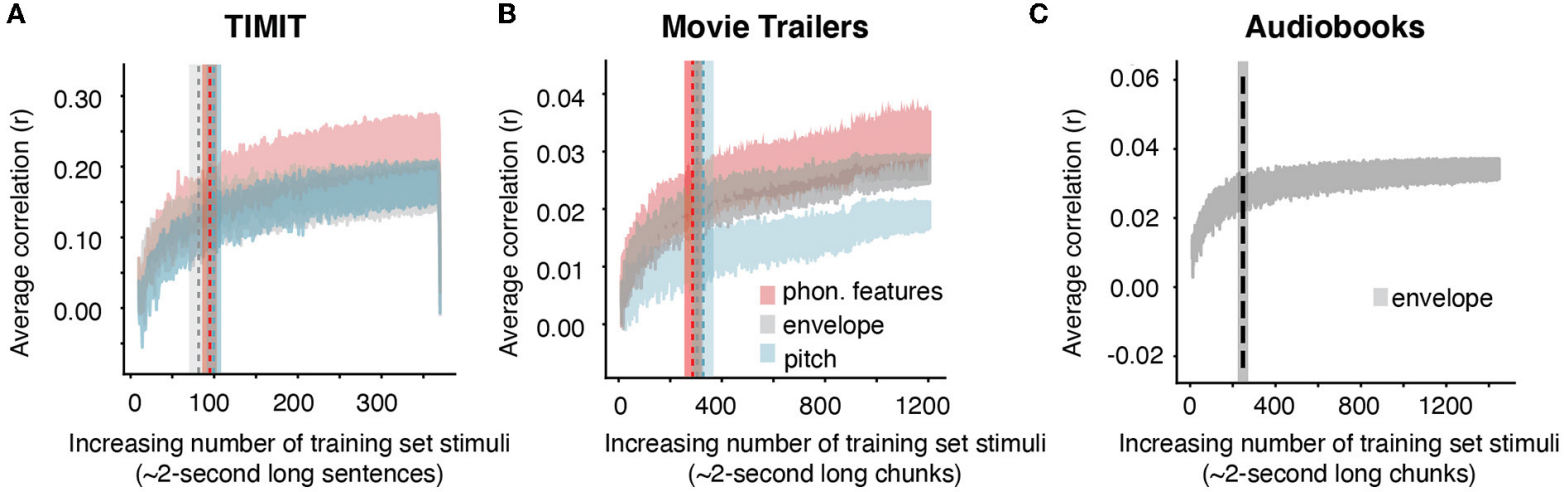
Average increasing correlation values based on acoustic or linguistic feature selectivity. **(A)** TIMIT average correlation values with standard error across all 16 EEG subjects for individual speech features (phonological features, envelope, and pitch) are shown as an average across all subjects when TIMIT training set sentences are iteratively added into the model. Dashed vertical lines and shading indicate the knee point and corresponding error bar for each individual model type. Despite different numbers of features and differing model complexity, a similar amount of data was required for each of these models. **(B)** Same as TIMIT but for movie trailers. Again, the knee point values did not differ significantly across models. **(C)** Audiobook average correlation across *n* = 19 EEG subjects using only the acoustic envelope as the feature representation (the pitch and phonological features were not provided in the dataset).

Our prior analysis showed that 96 sentences from TIMIT (192 s) or 565.76 s of movie trailers was where prediction performance for our full acoustic-phonetic model plateaued. However, what if an experimenter only wants to model neural responses to a specific feature or set of features, for example, the acoustic envelope? The structure of the stimulus itself is important in predicting responses to specific features, as certain features may require more training examples (Crosse et al., 2016). For example, models may require more training data for multivariate features, such as the spectrogram, but they may require less data for single features like word onsets. Still, even for single features, sampling the input space appropriately is important for building a robust model that will predict responses to unseen data. Having a diversity of voices with different pitch ranges would provide more robust model performance for a pitch-based model than including only a few sentences all from the same talker. To determine whether the amount of data needed varies depending on the stimulus feature of interest, we assessed the amount of training data needed for each individual auditory feature. As calculated from the grand average knee point, models predicting EEG from only the acoustic envelope required ~81 sentences (or 162 s of sentences based on the average length of each TIMIT sentence of 2 s) (Figure 2, gray shaded trace). However, models using phonological features and pitch required the same amount of data as the full auditory model (100 sentences or 200 seconds for pitch and 95 sentences or 190 seconds for phonological features) (Figure 2, red and blue traces). Additionally, when using the audiobooks from Broderick et al. (2018), predicting EEG data from just the acoustic envelope required 228 2-s-long audiobook chunks (or 456.92 s) of training set data. Despite a small numerical difference between the amount of data needed for the envelope model and other models (in comparing TIMIT and movie trailers only), these differences were not significant. Table 1 shows the fixed effects from the LME model for both TIMIT and movie trailers. In a linear mixed effects model with the knee point from each model type as a main effect and subject intercept as a random factor, the effect of model type on knee point was not significant for TIMIT nor movie trailers, suggesting that variable amounts of data based on feature type are not needed, at least for the features tested here (*p* > 0.05). Of note, the audiobooks only included the acoustic envelope as the low-level speech feature, so that is what was tested here.

**Table 1:** Fixed effects for each stimulus from LME.

	**Estimate**	**Std. error**	**df**	***t*** **-value**	**Pr (>|t|)**
**TIMIT**					
(Intercept)	96.125	10.481	45.909	9.172	6.02e-12^***^
Envelope	−15.062	12.224	45.000	−1.232	0.224
Phn. features	−1.625	12.224	45.000	−0.133	0.895
Pitch	3.563	12.224	45.000	0.291	0.772
**Movie trailers**					
(Intercept)	282.875	30.884	42.188	9.159	1.38e-11^***^
Envelope	22.188	34.525	45.000	0.643	0.524
Phn. features	4.688	34.525	45.000	0.136	0.893
Pitch	45.875	34.525	45.000	1.329	0.191

The intercept represents the effect of the combined model, whereas the relative effects of the envelope, phonological features, and pitch are shown. None of the individual models significantly differed in terms of knee point when controlling for subjects as a random factor.

The

*** symbol indicates the statistical significance at *p* < 0.001.

Lastly, the number of EEG channels varied between the datasets used in this study. For the movie trailer and TIMIT datasets, we used 64 channels, while the audiobook dataset from Broderick et al. (2018) used 128 channels. We conducted an independent sample Wilcoxon rank sum test to compare the knee point calculations between 32 versus 64 channels (TIMIT and movie trailers) as well as 64 versus 128 channels (audiobooks). We found that the number of EEG channels used for assessing the minimum amount of data for each stimulus set was not statistically significant (audiobooks: *W* = 0.146, *p* = 0.88, TIMIT: *W* = 0.283, *p* = 0.77, movie trailers: *W* = 0.13, *p* = 0.90).

### 3.3. How do receptive fields stabilize over time?

Our previous analyses assessed the correlation between actual and predicted EEG from the encoding models with increasing numbers of training set stimuli. Although this can show a qualitative stability of model weights, it does not show whether the fitted weights themselves are similar and stable when using more or less training data. Such an analysis is critical for researchers who wish to interpret receptive field tuning or structure. Thus, we assessed how the weights stabilize over time when adding more training data. From previous work, the assumption when building a model is that the relationship between the neural response and the stimulus feature will stabilize over time (Holdgraf et al., 2017). However, such an assumption may not always be the case because of varying signal-to-noise ratios for certain subjects or even factors such as participant attention or adaptation to the stimuli (Gibson et al., 2022). Calculating the stability of the model performance by observing changes in the weights can provide a metric for how robust the model performance is and determine how much training data is proven useful on an individual subject basis.

**Figure 3:**
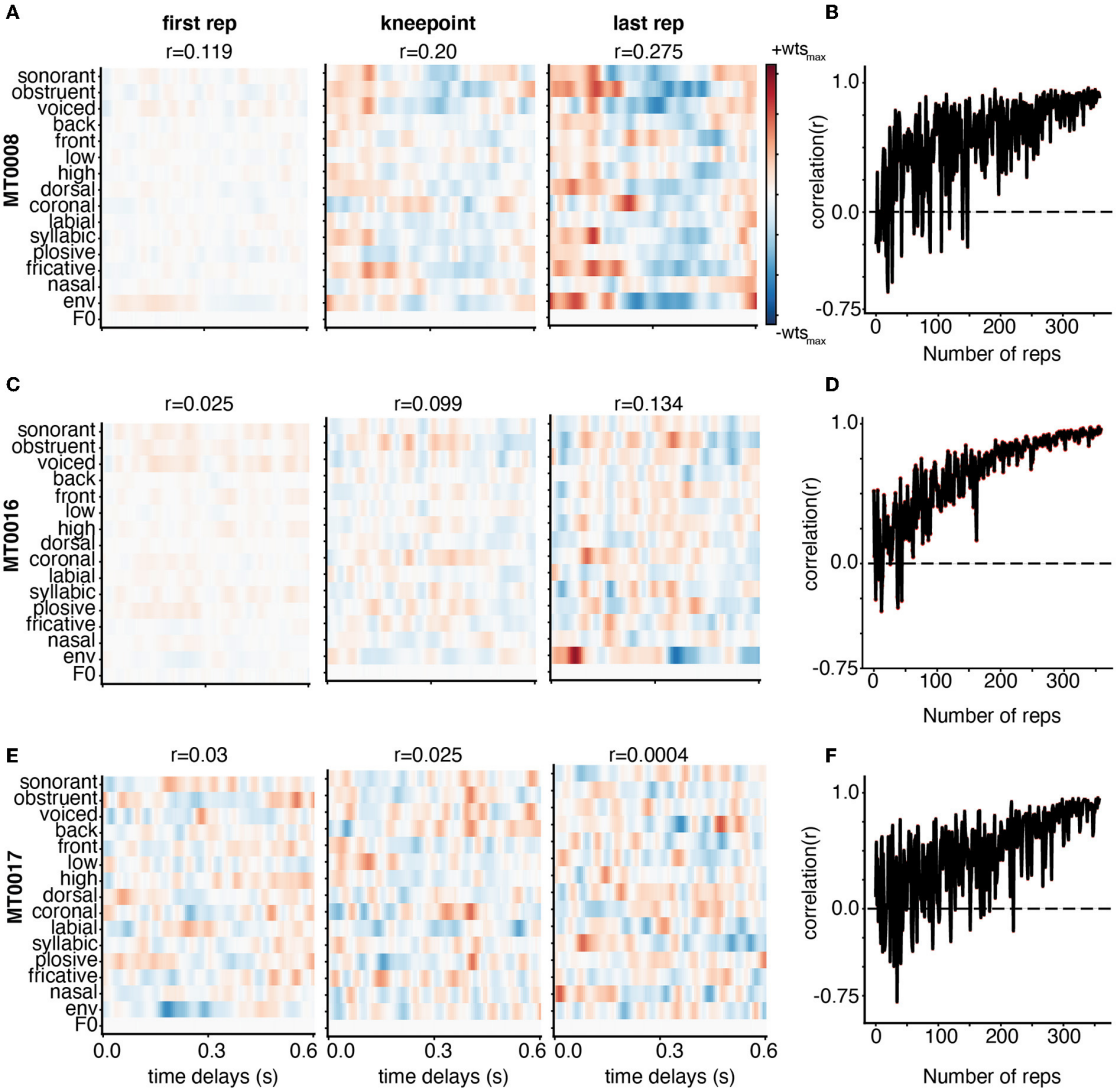
Weight stabilization for three subjects with varying model performance using TIMIT. Subjects MT0008 **(A)**, MT0016 **(C)**, MT0017 **(E)** are shown as examples of the best, average, and poor encoding models based on the correlation between predicted and actual EEG for held out data. The model performance correlation values are reported above the corresponding receptive field. Weights are shown from the encoding model based on the first repetition (left), knee point value for that participant (middle), and last repetition (right panel). For all subjects, the weights are plotted for all auditory features across time delays: 0–0.6 s. In the best and average participant, the model weights between the knee point and the last repetition appear similar with clear structure. On the other hand, the poorly fit participant (MT0017) has no discernable structure in the receptive field at any repetition. **(B, D, F)** Adjacent weights for the full auditory model for each subject were correlated with each other and plotted across the number of repetitions. This curve shows a general trend toward stabilization of receptive field structure as repetitions are added to the training dataset.

Here, we demonstrate the weight stability for three subjects with varying levels of model performance as defined by the linear correlation between predicted and actual EEG data for each iteration of adding training data. Participant MT0008 (Figure 3A) had the best performance, MT0016 (Figure 3C) had middle model performance, and MT0017 (Figure 3E) had the worst model performance. The performance value was determined by using the full model (combination phonological features, the acoustic envelope, and pitch) to obtain the overall average correlation value. As such, the overall correlation (*r*-value) for TIMIT between predicted and actual EEG data for the best subject, average subject, and worst subject model performance are as follows: MT0008 (*r* = 0.23), MT0016 (*r* = 0.11), MT0017 (*r* = 0.002). The structure of the receptive fields emerged as training data were added, starting with a relatively unrefined, low magnitude weight matrix with only one repetition of training data (Figures 3A, C, E). As more training data were added into the model, spanning from knee point until the final repetition, the receptive fields become more prominent, showing more feature selectivity across time delays. In the case of subjects with average model performance (MT0016 in Figure 3C, middle panel), the receptive field weights demonstrate sparse selectivity of acoustic and linguistic features across time delays in comparing the first repetition of training data compared to the knee point. Finally, subjects that had poor model performance had relatively noisy receptive fields across all iterations of adding training data (Figure 3E, bottom panels). Thus, varying signal-to-noise ratio may play a role in investigating how the weights change with increasing amounts of training data.

Finally, to quantify how the weights stabilized with increasing amounts of training data, we performed a correlation analysis between weights from subsequent additions to the training set (e.g., when including 100 sentences vs. 101, or 101 sentences vs. 102) (Figures 3B, D, F). If the receptive field has similar structure as data are added to the model, we should see that reflected in a stabilization of the correlation between the weight matrices on subsequent iterations of model fitting with added training data. We found that the correlation value (*r*-value) greatly fluctuates when fewer training set sentences are included, but eventually stabilizes, generating high correlation values between subsequent weight matrices with additional training data. These findings complement the receptive field data shown in Figure 3A, C, E in which the correlation plots between each adjacent weight increases until finally hovering close to the 1.0 correlation value. The higher the correlation values between adjacent weights (comparing each repetition to the next), the more correlated and more stable the weights are. These correlation plots also showcase how noisy the receptive field structure may be from one training iteration to the next. Participants with better model performance showed stronger and more stable feature selectivity in the receptive field with increasing amounts of training data, as shown both by visually similar weight matrices and stabilizing pairwise correlations.

**Figure 4:**
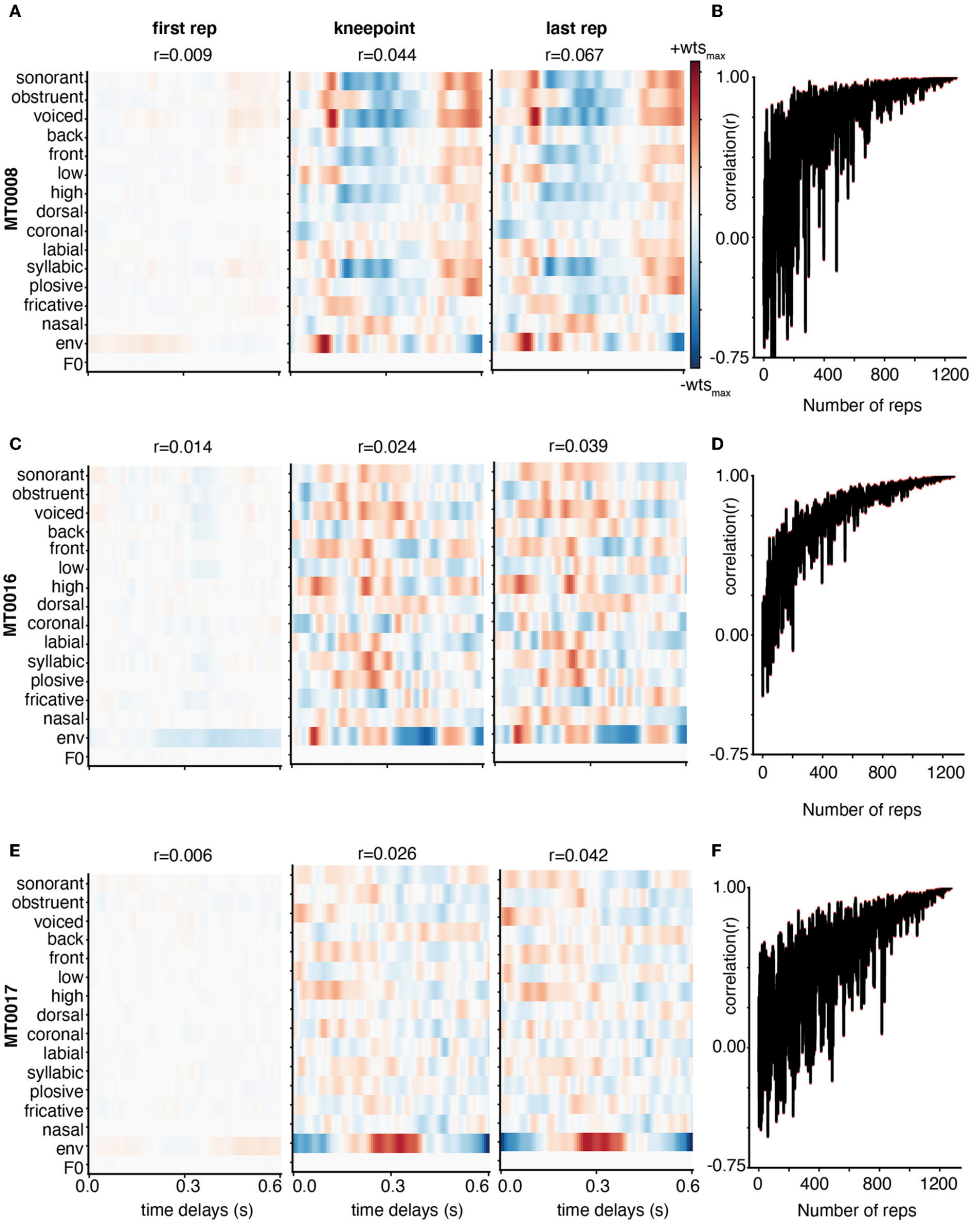
Weight stabilization for three subjects with varying model performance using Movie Trailers. **(A, C, E)** are the same as described in Figure 3, but instead use the same subjects to show weight stability across the number of repetitions for movie trailers. Here, the receptive field structure appears to emerge at the knee point and final repetition in all three cases. **(B, D, F)** show the correlation of the receptive field at repetition N with the receptive field at repetition N-1. At first, receptive fields are relatively unstable as reflected by oscillating correlations, but then they stabilize as data are added.

We find that the results using movie trailer data are comparable to those from TIMIT. When adding more training data into the encoding model between the first repetition to the knee point, and to the final repetition, the receptive fields show more selectivity in subjects with better model performance (Figures 4A, C, E). The best, average, and worst model performance from the movie trailer stimuli is also the same: MT0008 (*r* = 0.05), MT0016 (*r* = 0.04), MT0017 (*r* = 0.03). In subjects where the model performance was average or weak, the receptive fields (Figures 4A, C, E) are relatively noisier due to the possibility of varying SNR levels between subject recordings. Similar to the TIMIT analysis, we also quantified how the weights stabilized between subsequent chunks of adding 2-s-long training set data (Figures 4B, D, F). We find that computing the correlation between adjacent weights for the movie trailers asymptotically rises when adding more training set data until the correlation value approaches 1.0.

## 4. Discussion and conclusion

Encoding models are widely utilized to understand how the brain processes continuous and naturalistic stimuli (Crosse et al., 2016; Holdgraf et al., 2017). Here, we expanded upon an analysis from previously collected data in our laboratory (Desai et al., 2021) and a publicly available dataset (Broderick et al., 2018). A frequent question that is posed when building these sorts of models is “how much data is enough?” Ultimately, this question is dependent on the type of stimuli used and the types of feature selectivity one is interested in investigating. This analysis highlights the use of several different naturalistic stimuli: continuous speech sentences from the TIMIT speech corpus (Garofolo et al., 1993), audiovisual children's movie trailers, and audiobooks. We show that the stability of models can be assessed for each of these stimulus types, and that for low-level acoustic models the data requirements are fairly similar. This paper provides a necessary comparison by investigating how the choice of stimulus and stimulus features ultimately impacts the amount of data needed to build a robust encoding model to understand if the data collected are usable given a minimal amount of training data. Our results suggest that, at least for the models tested, the effect of stimulus representation (phonological features, envelope, or pitch) is weaker than the effect of using a different stimulus altogether. Still, with each stimulus set we were able to show a convergence of receptive field structure and model performance in significantly less time than a typical neuroscience experiment.

### 4.1. Caveats and limitations

Overall, the results from this study can only provide a suggestion as to how much data is needed for EEG encoding models, and absolute timing requirements should always be inspected and validated before assuming a specific amount of time is sufficient for a given purpose. First, the amount of data required to acquire robust results and stable receptive fields is specific to the stimuli (TIMIT and movie trailers) we used in our previously published work (Desai et al., 2021) and audiobooks from an open EEG data set (Broderick et al., 2018). Researchers using other natural speech stimuli should consider collecting pilot data and then assessing the minimum amount of data required for their specific experiment.

While the current study assesses the amount of data an experimenter would need to build a natural speech experiment using EEG and acoustic and phonetic information, we were unable to assess effects for higher order features. Prior work has tracked neural responses to natural speech using semantic information as a feature input in fMRI and EEG (Huth et al., 2016; Broderick et al., 2019) as well as higher frequency information to continuous speech (Kegler et al., 2022). While incorporating semantic information in EEG encoding models for natural speech experiments may be a challenge due to the large high temporal resolution and thus large number of time delays, researchers who seek to build natural speech experiments using fMRI may consider a similar bootstrapping analysis to assess the amount of data they may need using such higher order features. However, in principle our method should also work to determine how much data would be needed for both lower and higher-order speech representations. Lastly, the amount of training data required may be dependent on the aim of a particular research study. In our previous study (Desai et al., 2021) as well as the current, we used acoustic and phonetic features to fit mTRFs to predict neural activity from TIMIT and movie trailer stimuli. Similarly, we added data from Broderick et al. (2018) and used the envelope. However, it is important to note that the Broderick EEG data set contained other speech feature representations such as word onset. Therefore, using other higher-order features may generate a different knee point.

The results discussed above show that a correlation knee point and thus minimal amount of data may be determined using our method on multiple datasets. However, there are other potential caveats that researchers should consider. For example, one may be concerned about the impact of using different EEG systems, the number of EEG channels used in analysis, the EEG cap brand, the signal-to-noise ratio in calculating the knee point for each subject, and the participant's engagement with the stimuli during the experiment.

Regarding EEG systems, researchers may choose to use either an active or passive system, with passive systems typically showing higher impedance. Saline-based electrode setups may also have the additional danger of changing impedance over the experiment due to the pads drying out. Here we used an active BrainVision system with EasyCap caps. In Broderick et al. (2018), the researchers used a BioSemi-128 channel system. We found similar results for these two systems in terms of knee point, but overall correlations for the models differed, likely due to differences in averaging over the test set. For those using passive systems, they may first consider replicating the analysis here on a larger dataset to determine if the same principles hold. Similarly, we do not believe that the cap brand would matter, however using an active vs. passive system may make a difference in terms of the overall signal to noise ratio. Most importantly, researchers should be sure that the caps used fit well and are placed snugly on the participant's head to maximize data quality.

As evidenced by our analyses across multiple participants with varying data quality, signal-to-noise ratio of the data may be an additional consideration in determining amount of data needed. To investigate this in our data, we used previously calculated noise-ceiling corrected correlation values, which represent the maximum possible correlation that could be observed for a given dataset given the underlying trial to trial noise (Desai et al., 2021). We then used these values to compare against the knee point for each stimulus. There was no significant correlation between the normalized/noise-ceiling corrected correlation values from the knee point. Thus, at least for this measure, the SNR did not play a major role for our TIMIT and movie trailer stimuli. We were unable to test the effects of SNR with the knee point calculation for the audiobook dataset as stimuli were not repeated.

Regarding participant engagement, we found that we needed more movie trailer data and audiobook data compared to TIMIT, even though participants perceive these stimuli as more engaging. The movie trailers may require more data because of the acoustically rich background information that co-exists with speech information. From our previous work (Desai et al., 2021), we calculated the speech-only information which occurred 34% of the time, speech with background sounds (e.g., sound effects and music), which occurred 35% of the time, and the background sounds only occurred 18% of the time. From this information, we believe that more data would be required for stimulus sets which involve other sounds in addition to a target speech stimulus.

Finally, an additional caveat is that all EEG participants from in Desai et al. (2021) and Broderick et al. (2018) were sampled from healthy population groups (no reported hearing loss, normal or corrected-to-normal vision, native English speakers, and no history of neurological disorders). Thus, generalizing the knee point calculations for the specific stimuli used in this study to clinical or pediatric population groups may not hold true and more data may be needed to achieve robust and stable receptive fields.

### 4.2. Final recommendations and key points

The field of auditory and computational neuroscience is now leaning more toward incorporating more naturalistic stimuli to generate ecologically valid experimental paradigms. While our previous paper (Desai et al., 2021) focused on examining if it was possible to replace continuous speech sentences (TIMIT) with stimuli that are more engaging, we wanted to better understand how much data we would need to obtain robust results. A common complaint with most psycholinguistic or neuroscience experiments is that they are long and boring. Experiments can be anywhere between 1 and 2 h long, requiring the participant to sit still and sometimes requiring them to listen to the same stimulus over and over. Thus, we hope to provide researchers in the field with some suggestions as to the type of stimuli we used and how much data are needed when building future studies. Additionally, we hope that this work provides information on the amount of data needed if one were interested in encoding selectivity of specific speech features (phonological features vs. pitch vs. acoustic envelope) or a combination of multiple lower-level speech features. For these measures, the data requirements appear to be similar, but researchers should always pilot on their own stimulus set before assuming how much data may be needed. We want to highlight a few key points from our study and the analysis conducted in this paper:

- From our results, we found that on average, 192 s of TIMIT data and 565.76 s of movie trailer data are needed from our dataset. Our entire EEG session per subject lasted close to 1.5 h, however our results suggest that the full original recording time is not needed to achieve the knee point.
- We found that 495.47 s of audiobook data were needed using the acoustic envelope as opposed to the original 1-h long task from Broderick et al. (2018). This analysis shows that our method can be applied to other datasets with similar results.
- To figure out how much data we needed for specific feature types for TIMIT, we found the following: 81 sentences (162 s) for the acoustic envelope, 95 sentences (190 s) for phonological features, and 100 sentences (200 s) for pitch, were needed on average across all 16 subjects. Despite these smaller numeric differences in how much data were needed to achieve a knee point, the differences were not statistically significant. Researchers might choose to err on the side of at least 5–10 min of training stimulus presentation for all of these task types. This assumes a test set size of 10 TIMIT sentences repeated 10 times, which remained constant in our analyses.
- For movie trailers, based on individual features, we found the following: 610.12 s for the acoustic envelope, 575.12 s for phonological features, and 657.5 s for pitch, were needed on average across all 16 subjects. This assumes a test set size of 271 s (2 movie trailers) in the event where most of the subjects listened to and watched both test set trailers. In the case where only one of the trailers were heard out of the test set, the duration of the single movie trailers was 136 s.
- While the knee point calculation does provide a quantitative measurement of the minimum amount of data overall, the correlation value still does continue to increase when adding more training set data for all three of stimuli described in this manuscript (TIMIT, movie trailers, and audiobooks).
- Identifying the receptive fields for subjects with good, moderate, and weak model performance provided more insight on how feature selectivity changed with having more data. Overall, the difference in correlation performance between the minimum number of trials (knee point) for both stimuli and the full dataset (~1.5 h of collected EEG data) had a smaller change in percentage (33.5% increase for TIMIT and 36.8% increase for movie trailers across all subjects). In comparison, the percent improvement between the first repetition to the knee point was greater for both stimuli: 62.5% increase for TIMIT and 236% increase for movie trailers across all subjects. Thus, in situations when the time to collect data may be limited, the percentage change suggests a substantial increase in average correlation value from the encoding model compared to the percentage change between the knee point and final repetition.

Generally speaking, more trials of data help achieve a higher signal-to-noise ratio for an optimal result. In an ideal world, collecting more data is better. However, including more trials in an experiment can ultimately be detrimental due to participant fatigue in both healthy subjects and clinical populations. Having fewer trials with better quality data, in which the subject does not move or fall asleep, can ultimately improve the data quality and also allow experimenters to collect data from a more heterogeneous population as opposed to fewer individuals. Additionally, there may be instances where smaller datasets are unavoidable due to time constraints when working with clinical populations such as those with hearing loss who may be easily fatigued or even pediatric populations, who may not be able to sustain attention for long periods of time. Finally, certain populations may prefer specific stimuli over others, such as stimuli that are more entertaining or engaging to listen to. In those cases, identifying the amount of data for a specific stimulus type becomes crucial to collect high fidelity and clean data, and to avoid discarding data that could be used in an analysis.

## Data availability statement

The datasets for this study are available upon request from the corresponding author. The code can be found in the following GitHub repository: https://github.com/HamiltonLabUT/dataset-size-considerations. The DOI for the repository is: https://zenodo.org/badge/latestdoi/584916596.

## Ethics statement

The studies involving human participants were reviewed and approved by The University of Texas at Austin Institutional Review Board. The participants provided their written informed consent to participate in this study.

## Author contributions

MD and LH designed the study, contributed to the experimental design, analyzed the data, prepared the figures, and wrote the manuscript. MD collected the data and performed all analysis. AF pre-processed the raw EEG from open audiobook dataset. All authors have read and approved of the manuscript.

## Supplementary material

Supplementary Figure 1 Feature comparison of the movie trailer stimuli that were presented to the EEG participants. A total of 23 movie trailers were presented. For some of the trailers (e.g., Big Hero 6, Ferdinand, Incredibles, The Lego Ningajo Movie), different variations of the trailer were shown. **(A)** Total duration of each movie trailer in seconds. **(B)** Heat map shows the total number of phonological features across all of the movie trailers. As expected, the sonorant and obstruent phonological features have the largest count for all trailers. A few trailers included very little speech (e.g., “bighero6-tlr1”). **(C)** Heat map shows the total count of binned pitch between the frequency ranges on the y-axis for all movie trailers. The pitch range between 51 and 85 Hz has the largest count across all trailers. **(D)** The average power spectrum for each movie trailer plotted across frequency based on the original audio sampling rate of 16 kHz.

Supplementary Figure 2 Individual and average correlation value for audiobook data. A total of 19 EEG subjects were used for the analysis in which they listened to an audiobook. For the audiobook data, each gray line shows how the correlation values change for an individual subject when adding each additional 2-s chunk of audiobook training set data. The average increasing correlation value of all subjects is shown in green with standard error. The dashed vertical line at 247 2-s-long chunks (495.47 s) indicates the average knee point across all subjects (gray shading shows standard error of the mean).
