# Supplementary figures and images for: Dataset size considerations for robust acoustic and phonetic speech encoding models in EEG
Source: Front Hum Neurosci. 2023 Jan 20;16:1001171. doi: 10.3389/fnhum.2022.1001171 (PMC9895838; doi:10.3389/fnhum.2022.1001171)

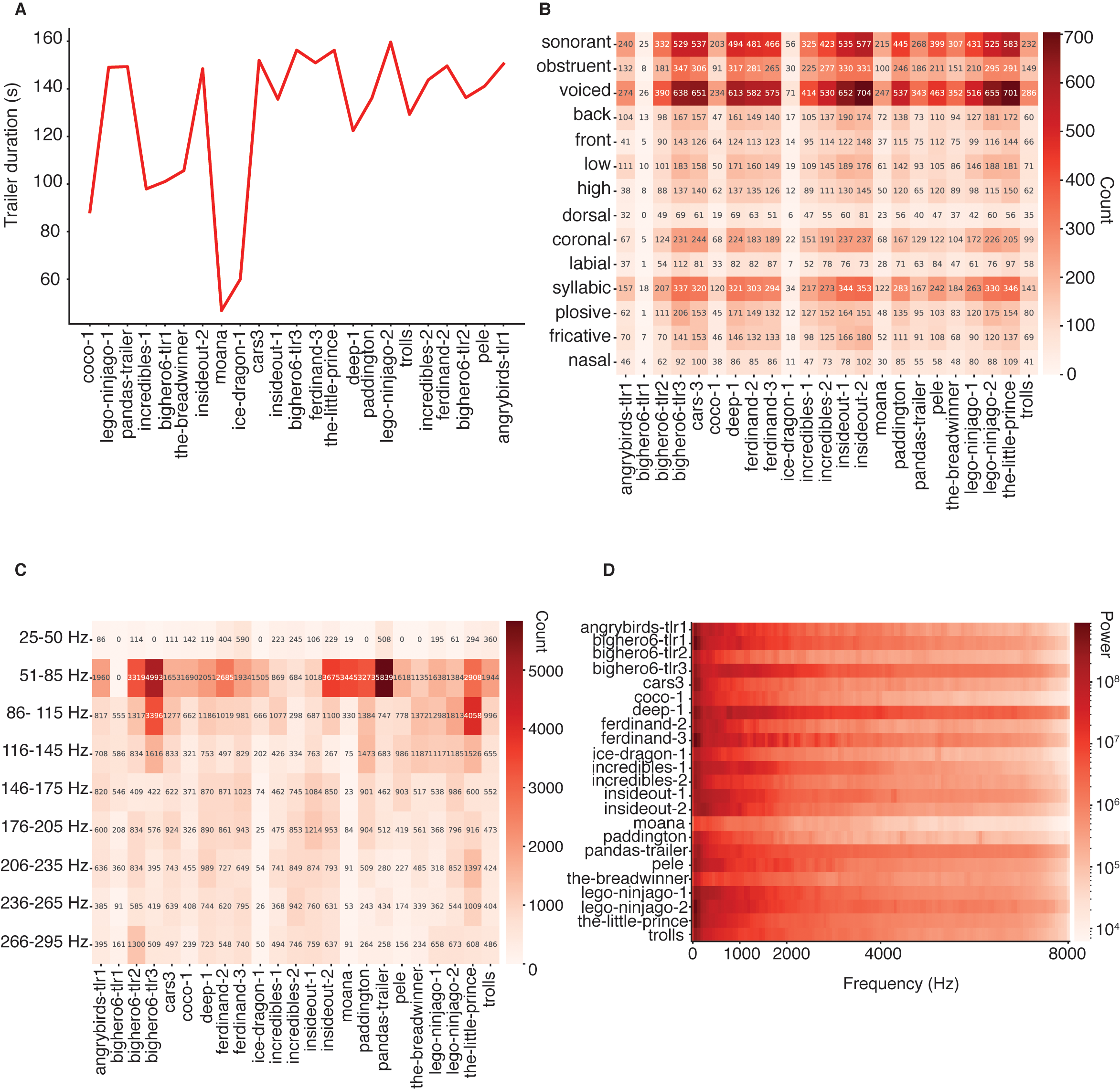

Supplement: Supplementary Figure 1 — Feature comparison of the movie trailer stimuli that were presented to the EEG participants. A total of 23 movie trailers were presented. For some of the trailers (e.g., Big Hero 6, Ferdinand, Incredibles, The Lego Ningajo Movie), different variations of the trailer were shown. (A) Total duration of each movie trailer in seconds. (B) Heat map shows the total number of phonological features across all of the movie trailers. As expected, the sonorant and obstruent phonological features have the largest count for all trailers. A few trailers included very little speech (e.g., “bighero6-tlr1”). (C) Heat map shows the total count of binned pitch between the frequency ranges on the y-axis for all movie trailers. The pitch range between 51 and 85 Hz has the largest count across all trailers. (D) The average power spectrum for each movie trailer plotted across frequency based on the original audio sampling rate of 16 kHz. [file Image_1.TIF]

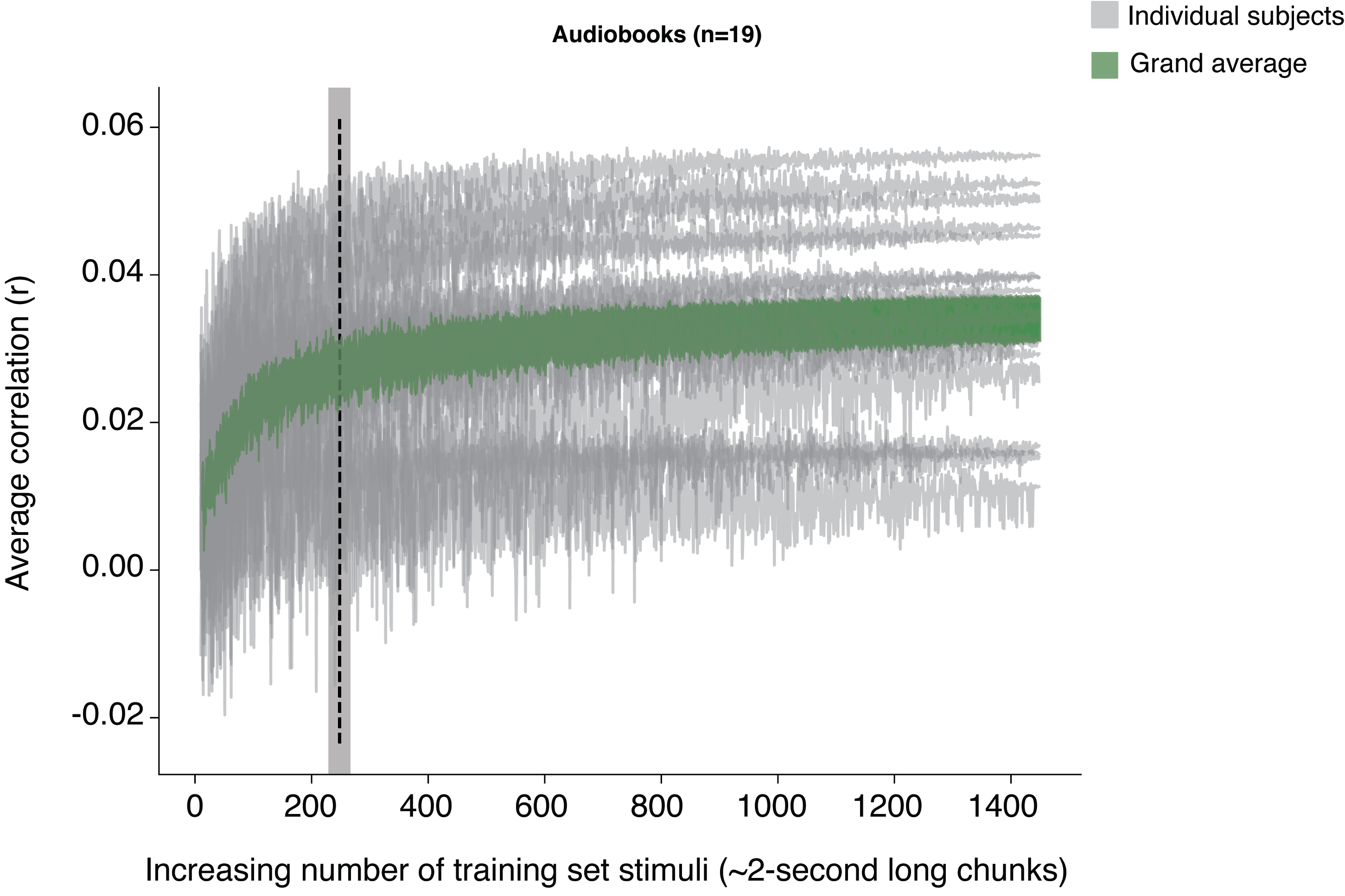

Supplement: Supplementary Figure 2 — Individual and average correlation value for audiobook data. A total of 19 EEG subjects were used for the analysis in which they listened to an audiobook. For the audiobook data, each gray line shows how the correlation values change for an individual subject when adding each additional 2-s chunk of audiobook training set data. The average increasing correlation value of all subjects is shown in green with standard error. The dashed vertical line at 247 2-s-long chunks (495.47 s) indicates the average knee point across all subjects (gray shading shows standard error of the mean). [file Image_2.png]
